# Inclusion of women susceptible to and becoming pregnant in preregistration clinical trials in low- and middle-income countries: A proposal for neglected tropical diseases

**DOI:** 10.1371/journal.pntd.0008140

**Published:** 2020-06-11

**Authors:** Monique Couderc-Pétry, Elisabeth Eléfant, Monique Wasunna, Alwyn Mwinga, Nilima A. Kshirsagar, Nathalie Strub-Wourgaft

**Affiliations:** 1 Pétry Medical Consulting, Suresnes, France; 2 Reference Center on Teratogenic Agents (CRAT), Armand-Trousseau Hospital, Paris, France; 3 Drugs for Neglected Diseases *initiative*, Nairobi, Kenya; 4 Zambia AIDS Related Tuberculosis Project (Zambart), Lusaka, Zambia; 5 Clinical Pharmacology, Indian Council of Medical Research, New Delhi, India; 6 Drugs for Neglected Diseases *initiative*, Geneva, Switzerland; US Food and Drug Administration, UNITED STATES

## Executive summary

The Drugs for Neglected Diseases *initiative* (DND*i*) aims to conduct research and development of new, improved, and patient-focused treatments for neglected tropical diseases (NTDs). DND*i*’s goal is to address unmet medical needs in low- and middle-income countries (LMIC) where they are needed using the best regulatory approval strategy for effective, safe, and affordable new treatments. When NTDs affect women susceptible to and becoming pregnant (WoSuP), a major concern, shared by DND*i*, is to ensure that there are available data to support the safety and efficacy of the treatments for these women and potentially for their babies.

WoSuP are female patients, sometimes very young, who have reached menarche and could become pregnant during treatment or during a clinical study or trial.

For several decades, there has been reluctance to include WoSuP in clinical trials because of the potential risk of exposing the fetus to investigational drugs. This has led to neglecting physiological differences between WoSuP and other adult populations.

This trend is, however, changing with arguments in favor of the ethical need to have appropriate data on new treatments in any population including WoSuP before regulatory approval. One of the systematic mitigating precautions taken to allow recruitment of WoSuP in preapproval clinical trials is the use of contraception. However, contraception is not always widely available, culturally allowed, followed, or effective in preventing pregnancy. Women might become pregnant during a clinical trial of a new treatment. The purpose of this paper is not to discuss the inclusion of already identified pregnant women but to envisage how to include WoSuP and provide them with access to innovative drugs, while acknowledging and mitigating the risk that they might become pregnant during the trial.

Understanding the existing limitations for the participation of WoSuP in clinical trials, DND*i* suggests the use of appropriate measures to ensure that WoSuP are represented as far as possible in trials, in accordance with scientific and ethical standards.

This paper describes DND*i*’s proposal, which is based on a benefit and/or risk assessment of participation versus nonparticipation of this population in clinical trials. The primary objective is to provide adequate and robust evidence of efficacy and safety in this population, while also considering the risks related to the NTD and alternative treatment landscape, and balancing these with possible embryo-fetal harm. Whether to include WoSuP or pregnant women at the time of the recruitment and whether to continue inclusion of women who become pregnant during clinical studies represent different situations. A safe ethical framework for the recruitment of WoSuP in clinical trials of new drugs against NTDs is the focus of this paper.

## Problem statement

DND*i* is a patients’ needs driven, not-for-profit research and development (R&D) organization that develops safe, effective, and affordable medicines for NTDs afflicting millions of the world’s poorest people. Working with public and private partners, DND*i* conducts research and develops new and improved treatments for NTDs that are adapted to the needs of patients in LMIC. DND*i*’s R&D pipeline encompasses a full range of drug development, from early research and preclinical through to phase I, II, and III clinical trials for diseases such as African sleeping sickness, leishmaniasis, Chagas disease, filarial diseases, and mycetoma and also for neglected patients such as those with pediatric HIV and hepatitis C. DND*i*’s goal is to obtain regulatory approval based on robust data in all population subsets who need such treatments.

WoSuP during a clinical trial are seldom considered a specific population in literature concerning therapeutic clinical development, and there are no guidelines for this population. This population is comprised of women of childbearing potential with a negative initial pregnancy test and access (or not) to adapted, safe contraception provided by the sponsor. In theory, these women should not become pregnant during the trial, but in practice several of them will start an “unauthorized” pregnancy.

NTDs may affect WoSuP either in the same way as other adult populations or there may be different interactions, and DND*i*’s major objective is to develop safe and effective treatments for these women while protecting any future, albeit unexpected, babies. The probability of a pregnancy is variable, but always present. This is the reason why in this paper the term WoSuP is preferred to “women of childbearing potential,” which is less explicit about the risk of pregnancy. In this paper, we do not address women who are already pregnant at the start of a clinical trial. Even if trial investigators do not anticipate pregnancies, sick women may become pregnant. In fact, the risk of unexpected pregnancy in a WoSuP during a clinical trial in a LMIC is particularly high. The mean age of the population is low in LMIC, and the number of pregnancies per woman is much higher than in industrialized countries; in addition, the risk of pregnant women suffering from NTDs in LMIC being exposed to drugs is high. Although the number of children per woman is lower for women included in clinical trials than in the general female population, pregnancies occur with higher frequency in clinical trials in LMIC [1].

Since DND*i* aims to develop treatments that can be rolled out to a large community, documenting the conditions under which the benefit and risk balance of new treatments may and should be studied in young women is essential. This paper is based on a literature review of different expert positions and contributes to supporting DND*i*'s ethical framework on the inclusion of WoSuP in clinical trials of new drugs against NTDs.

This framework is in full accordance with the protection of participants established in the Declaration of Helsinki [2]: “Medical research involving a disadvantaged or vulnerable population or community is only justified if the research is responsive to the health needs and priorities of this population or community and the research cannot be carried out in a nonvulnerable population. In addition, this population or community should benefit from the knowledge, practices, or interventions that result from the research. Consideration should also be given to ensuring that the community receives a fair level of additional benefits."

It is important to note that DND*i* is usually not the authorized marketing company and may not be responsible for postapproval or pharmacovigilance activities. The collection of data postapproval is certainly needed to improve the benefit and risk assessment of new drugs in field conditions, e.g., “real-life data,” adverse event spontaneous reporting, or cohorts. Education plans for women or availability of contraception after development are not within the DND*i* scope and are therefore not addressed in this paper. We review the situation for women with or without safe and effective contraception and a negative pregnancy test at inclusion and women who unexpectedly become pregnant during a trial. For both situations, this paper proposes algorithms to facilitate the decision to include and follow-up WoSuP in clinical trials.

## Historical background and context

For decades, historical justifications have been given for excluding WoSuP from clinical trials.

### Risks of exposing a fetus to investigational drugs given to WoSuP

It has long been considered dangerous for a WoSuP to participate in trials because of the potential risks for the embryo or the fetus [3]. The main rationale was the fear of unknown toxicity of the investigational drug, which could cause birth defects or developmental harm.

WoSuP are at high or higher risk of becoming pregnant during clinical trials in LMIC for the following main reasons:

- Poor access to contraception or insufficient compliance with contraception by the patient or her partner
- Contraceptive failure, even with good compliance; within the first year of use, the risk of unintended pregnancy ranges from 0.05% to 20% with perfect use [4]
- Interactions between contraceptives and concomitant treatments (anti-tuberculosis or HIV, antifungal, antiepileptic, and/or mental disorder drugs)
- Delay in efficacy of oral or injectable contraception (up to 4 to 7 days)
- Desire to have a child despite the investigator’s advice. Furthermore, when the investigational treatment has good efficacy, the improvement in health status of WoSuP may increase the willingness to become pregnant or the likelihood of this happening

Worries about embryo and/or fetal safety obviously loom large not only for researchers but also for WoSuP and their healthcare providers. These concerns could lead clinicians to undertreat or even not treat illnesses that persist, worsen, or emerge when a WoSuP becomes pregnant. Importantly, while research can tell us when drugs are unsafe, it can also reassure us when they are safe. There may also be concerns around budgeting for the unexpected, nonnegligible costs of taking care of potential pregnancies and complications to the mother or fetus and insurance costs (liability) in case of any unwanted outcome [1].

### Physiological characteristics of WoSuP

Physiological differences between WoSuP and adult men, or infertile or postmenopausal women, can have some impact on the safety and efficacy of drugs through differences in kinetics or pharmacodynamics [5]. Heterogeneity goes beyond differences between men and women as women’s reproductive cycle phases and concomitant hormonal effects are great contributors to these differences [6]. These differences can also lead to an increased variability of clinical trial results. Studying a homogeneous population (i.e., limited to men) decreases the sample size needed to detect a significant treatment effect or adverse effects. This might be justifiable for “first-in–human” studies.

For those WoSuP who become pregnant during a trial, physiological changes will affect further drug pharmacology with, in particular, changes in total body weight and body fat composition, increases in plasma volume and cardiac output, changes in regional blood flow, increases in glomerular filtration rate, alterations in gastrointestinal mobility, decreases in albumin levels, and changes in hepatic enzyme activity and CYP450 isoenzyme drug metabolism [7–10]. These differences can lead to an increased variability in clinical trial results.

### Legal and regulatory environment

Institutional review boards (IRBs) or research ethics committees (RECs) oversee the protection of participants and consider liability issues. To protect the rights and welfare of human subjects participating in clinical trials, the IRB and RECs must ensure that research participants are properly informed of the potential risks of treatments evaluated in clinical trials [3]. Strikingly, there is no legal framework that prohibits the inclusion of WoSuP in clinical trials.

In 1977, the United States Food and Drug Administration (FDA) issued a guideline recommending that females of childbearing potential be excluded from the earliest clinical studies. It suggested that these women could participate in clinical studies if adequate information on efficacy and relative safety was obtained during early Phase II and if animal reproductive toxicology studies were completed and no adverse effect on the fetus was found. However, the use of contraception to avoid pregnancy during studies was not addressed [11].

### Ethical aspects

The low level of inclusion of young women in clinical trials [12] limits the availability of relevant scientific information about treatments in this population, even though, ethically, they have the same right to be treated with drugs deemed effective and safe based on evidence. Exclusion deprives women as a group of the benefits of new knowledge derived from clinical trials and can be considered an insult to their right to self-determination [13].

Most Phase II and III trials are expected to show that a drug is effective for a given medical condition. Since participants in the test arms of trials may benefit significantly, especially where no other treatment exists, excluding WoSuP puts them at an unfair disadvantage in terms of health and well-being [5,14].

In addition, operationally, restricting trial inclusion to men or postmenopausal women slows the rate of recruitment considerably and may delay the availability of helpful drugs against NTDs, particularly in the case of less prevalent diseases.

Participating in clinical trials could also improve the health of WoSuP, with more follow-up, consultations, laboratory testing, and better information, in a similar way to children being “safer” in a trial than when receiving drugs used off-label [15].

## Evolving landscape

A new trend is emerging, however, due to increasing advocacy for appropriate representation of women in clinical trials. For example, in some recent HIV clinical trials conducted by the US National Institutes of Health, the percentage of women was higher compared to men [1].

There is still significant progress to be made. Several recent US studies evaluating gender analysis in randomized clinical trials published by nine major medical journals showed that in trials for diseases without sex-specific prevalence, the average enrolment was only 37% women. Furthermore, 64% of the trials did not provide results by sex and did not justify why the influence of sex on their findings was ignored [16].

Similar findings have also been recently described for LMIC. An analysis of the Clinical Trial Registry-India showed that, in 2015, 48 of the 1,124 trials registered involved only men, 128 only women, and 948 both men and women. However, these numbers provided false reassurance, as an analysis published in PubMed showed that 19% of 134 clinical trials performed in India over the past 5 years did not mention gender and, of those that did, most had fewer than 50% women. The lack of research including women was also reflected in the lack of data in the package insert: Of 26 new drugs approved in India in 2015, over half had no information relating to women due to lack of data [17].

Some now argue that the potential for becoming pregnant during a trial should not continue to be used as a reason to preclude or limit participation of young women in research [13].

It is possible both from an ethical and regulatory standpoint to conduct clinical trials with WoSuP if:

- Women are given prior and complete information on the potential risks and benefits of treatments for themselves, their unexpected but potential pregnancy, any resultant offspring, and their fertility
- Research is relevant to the healthcare needs of WoSuP
- Information has been obtained from reliable results in animals, mainly in terms of risks for reproductive toxicity and genotoxicity

Other factors that need to be analyzed and integrated in the benefit and risk assessment include the epidemiology of the disease, the probability of WoSuP exposure, the type of disease (acute or chronic, seriousness), and, if left untreated, the impact of the disease on an unexpected pregnancy [18].

## Conditions for involving WoSuP in clinical trials

### Prevention of pregnancy through contraception during clinical trials

Contraception is a pregnancy-mitigating factor when recruiting WoSuP. In 1993, the FDA revoked its 1977 guideline, acknowledging that fetal exposure could be prevented by appropriate protocol design and contraception [16,19]. The 1993 guideline recommended that the drug effectiveness and adverse effects be analyzed by gender and that pharmacokinetics be characterized in males as well as females [11]. However, implementation was not systematic.

Despite all efforts to avoid pregnancy during trials, in particular by providing complete information to women, the risk of unexpected pregnancy remains. Pregnancy tests should always be performed before inclusion into a trial and female contraceptives and/or condoms should be provided [20,21]. In practice, contraception may be initiated at the time of the visit, which may not correspond to the cycle start or equally be initiated at the start of a cycle but after Day 1 of the study treatment. This undermines the efficacy of contraception. The time required for female hormonal contraception to take effect must be taken into consideration, with the possible use of condoms in the meantime. However, this is rarely done.

A variety of sensitive issues and ethical concerns are raised when providing contraceptives. In some contexts, delivering contraceptives to women may be unfeasible or seen as culturally unacceptable, particularly to adolescents or unmarried women. In this case, the unwanted result will be to continue to exclude these women from drug development [22,23]. The provision of free contraception may be perceived by some as a monetary incentive to participate, and it carries subtle ethical implications; research has shown that monetary incentives have a greater impact on women than on men on their willingness to participate in research. Additionally, religious or cultural objections may prevent some women from agreeing to participate in a study requiring the use of any contraceptive method [24,25].

When including adolescent WoSuPs, it may be useful to implement programs including pregnancy prevention and educational-career motivation. Such programs, aimed at youths in communities, may include mentoring to teach, counsel, and provide information to improve their health, education, career, and social outcomes. Educational-career programs provide knowledge to young women and men, enabling them to make well-informed decisions [22,26].

Finally, as women are often influenced by people around them, such as their husband or the head of the village, the choice of method of contraception should not be imposed because this negatively impacts women’s reported willingness to participate in research [19,27].

## Preclinical and clinical reproductive and toxicology package for early clinical development with WoSuP

Some recommendations have been published to improve the predictability of animal toxicity results. After the thalidomide disaster, several international regulations have been implemented to improve the evaluation of the impact of new drugs on animal reproductive function prior to their use in humans: choice and number of species, studies of fertility, embryonic and fetal periods, peri- and postnatal (including nursing) periods, effects on the offspring, etc. These provisions are described in various guidelines of the International Council for Harmonisation of Technical Requirements for Pharmaceuticals for Human Use (ICH) used by regulators and industry across the world. The main guideline (M3) requires the study of the three major segments of the six successive stages of reproduction and development [28]. The recommended approach for animal studies is a combination of studies on fertility and early embryonic development, embryo-fetal development, and pre- and postnatal development in one or two species [14].

It is necessary to have some toxicokinetic data as well, since these data may indicate the need to use a different species or adjust the study design or dosing schedule. Human risk should be anticipated if anomalies such as growth retardation, malformations, or abortions are observed in animals at exposure levels similar to those for humans at the therapeutic dose [29]. The main situations are summarized in Table 1.

**Table 1:** Animal toxicity and human risk.

	Compound-related animal abortions or teratogenicity	Compound-related fetal toxicity (excluding malformations)
**Animal exposure level similar to human therapeutic** **dose exposure**	Human risk likely	Human risk cannot be excluded
**Animal exposure level several-fold higher than human therapeutic dose exposure**	Human risk cannot be excluded	Human risk unlikely

Other major findings are usually considered as predictive of increased risk of teratogenicity:

- Number of impacted animal species
- Similar malformations in several animal species [30]

It is imperative to ensure proper methodology in animal studies that allows identification of potential gender differences and identify whether such differences affect efficacy or safety of new drugs [23,25,31–33].

In accordance with European Medicines Agency (EMA) guidelines, WoSuPs can be included in early clinical trials without prior nonclinical developmental toxicity studies in certain circumstances [34]; these would include prior knowledge of the mechanism of action of the drug, its pharmacological class, the extent of fetal exposure, or the difficulty of conducting developmental toxicity studies in an appropriate animal model for example for some biologicals.

### Weighted assessment of risk

Sponsors, researchers, regulators, and IRB and REC members systematically weigh the potential risks of any proposed study against the benefits. There is an unavoidable need to take calculated risks and accept trade-offs for WoSuPs in relation to the risk of unexpected pregnancy. Indeed, even for drugs with known teratogenicity, calculated trade-offs may still be possible [14,33].

For example, the US Federal Policy for the Protection of Human Subjects regulations ask IRB and REC members to consider inclusion of WoSuP once prior research has been performed. Where scientifically appropriate, preclinical studies, including reproductive toxicology studies in pregnant animals, and clinical studies, including studies on nonpregnant women, should be conducted to provide data to assess the potential risks to both pregnant women and fetuses. Inclusion of young women may be initiated where there is either the prospect of direct benefit to a woman or fetus or when the risk is no greater than minimal [3].

The 2008 European Guideline on Risk Assessment of Medicinal Products on Human Reproduction and Lactation: From Data to Labelling [34] describes the process of integrating nonclinical and clinical data and highlights important considerations for the assessment of adverse reproductive and/or developmental effects in humans based on reproductive toxicity studies in animals and human clinical data. Further work on pregnancy guidelines is ongoing at the EMA.

### Evolving ethical aspects of WoSuP autonomy

In parallel with the evolution of the ethical need for robust evidence in WoSuP described above, it is fundamental to respect women’s autonomous decision-making [3,35]. This means that women must be allowed to make their own decisions about a potential teratogenic risk, including in settings without access to contraception or abortion. The consent discussion with WoSuPs should include a discussion on benefit and risks for mother and child and information about the option of withdrawing voluntarily and at any time from the trial if necessary and of, where legally permissible, terminating the pregnancy. In particular, if the pregnancy is not terminated, women should be guaranteed medical follow-up [13].

Informed consent given by the woman alone (as a competent adult) is required for her participation. In no case should the permission of a spouse, partner, or family member replace the requirement for the individual’s informed consent. However, in most countries, female adolescents are still considered legally incompetent to provide consent to participate in a clinical trial, and parental consent will be needed. In all situations, specific information should be provided, and the adolescent’s assent sought.

There are communities or societies in which cultural or religious beliefs place more importance on the fetus than on the woman’s life or health, and women may either feel forced to participate or refuse participation in research. Special safeguards should be set up to prevent undue inducement for WoSuPs to participate in research [13].

### When a WoSuP becomes pregnant during a clinical trial: Risk of exposing the embryo or fetus to investigational drugs

**Fig 1:**
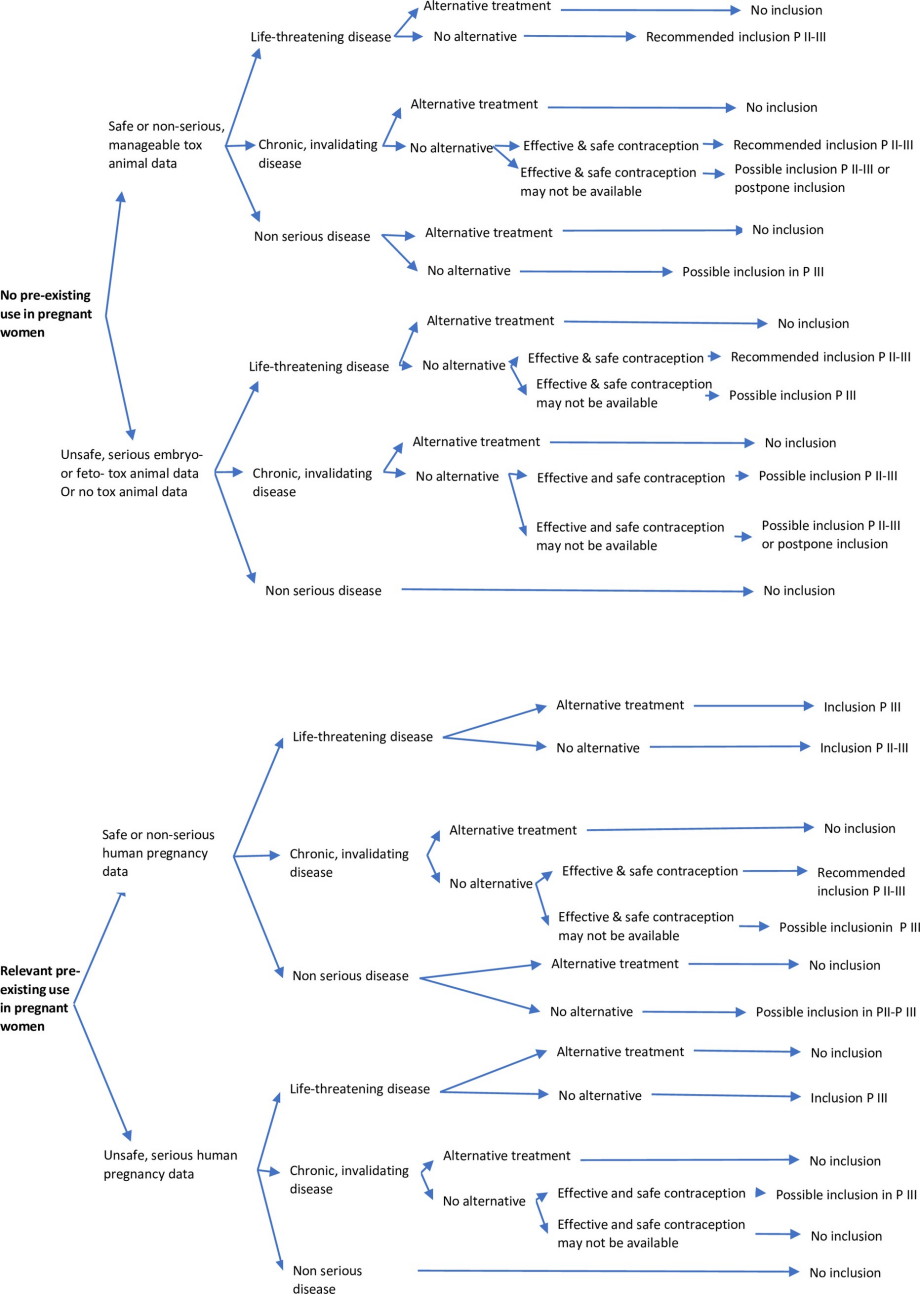
WoSuP inclusion in clinical trials, with availability of safe effective contraception a negative pregnancy test at inclusion, pregnancy test repeated at each visit, and access to safe abortion. “Safe or nonserious, manageable (toxicology data) effects” indicates not life-threatening, no major disability: for example, supernumerary ribs. “Unsafe, serious (toxicology data) effects” indicates life-threatening or invalidating: for example, cardiac, cleft lip, or neural tube defect. “Life-threatening disease” indicates immediate or rapid death: for example, serious malaria access. “Chronic, invalidating disease” indicates important impact on daily activities: for example, leishmaniasis. “Effective contraception” indicates accessible, suitable, and well-accepted, effective contraception. “No inclusion” indicates formal contra-indication to include WoSuP. “Avoid inclusion” indicates inclusion restricted to where no available treatment alternative. “Possible inclusion” indicates inclusion after balanced evaluation of pros and cons. “Postpone inclusion” indicates inclusion after the end of pregnancy. “Recommended inclusion” indicates encourage inclusion of WoSuP to increase the chance of treatment or cure. “Inclusion” indicates same risks from participation in the trial for WoSuP as for other populations. P, Phase.

It is not mandatory in all cases to withdraw a WoSuP who unexpectedly becomes pregnant during a clinical trial [36]. The different factors to consider for pregnant women in clinical trials are described in Fig 1; the case of a woman becoming pregnant during a clinical trial or being (unexpectedly) found to be pregnant after the inclusion are quite similar.

In fact embryos or fetuses are most frequently exposed to drugs outside clinical trials, when young women take medications and become unexpectedly pregnant or do not realize that they are already pregnant, particularly in the early weeks of pregnancy, which are the critical period for the occurrence of malformations. This may occur after the drug they take has been approved, and postapproval registries should always be set-up for the follow-up of exposed pregnancies, even if registries are difficult to manage in LMIC.

If a WoSuP becomes pregnant during a trial, the level of evidence available must be reviewed. When a new treatment is launched on the market, there are no preapproval studies or data from during human pregnancy, and therefore the only data available will be from animal studies of teratogenicity or fetal toxicity, but these data have variable predictive value [16,23].

Pregnant and breastfeeding women are considered a neglected population in many publications and guidelines on clinical trials. They are considered “therapeutic orphans,” similar to the term used for children. Significant efforts have been made to not exclude these women who become pregnant during clinical trials at the same time as keeping them safe. In a few recent cases, where the maternofetal consequences of the untreated disease are known to be serious for the mother, the child, or both (H1N1 influenza, Ebola, and Zika), the administration of approved treatments or drugs under development has been accepted in WoSuP [37].

These efforts are important for several reasons:

- Many infectious diseases, such as malaria, are highly prevalent in pregnant women in whom the disease can become serious and poses risks to both the mother and the fetus.
- There is a risk of vertical transmission of the disease to the fetus. For example, in Chagas disease, the parasitic infection with the greatest morbidity in Latin America, the congenital infection rate is between 1% and 10% [38]. Its prevalence is about five to six million people [39] with about 14,000 deaths annually. In pregnant women, the prevalence reaches 20% to 40% in some regions of Bolivia [40]. A similar risk has also been described for sleeping sickness.
- In contrast, several diseases, such as leishmaniasis and sleeping sickness, decrease the fertility rate of women, numerically reducing the risk of fetal consequences.
- Treatments often present safety issues for the whole population, such as antimony treatment for leishmaniasis. Some much needed drugs may be contraindicated in pregnant women. For example, nifurtimox and benznidazole used for Chagas disease are contraindicated during at least the first trimester of pregnancy.
- Women are frequently exposed to drugs during their pregnancy. In industrialized countries, 64% of pregnant women will be given at least one prescription [3]. The average female US citizen receives 1.3 prescriptions per pregnancy, and two-thirds of women use four to five drugs during pregnancy and labor [5]. Unfortunately, similar data are not available for LMIC.

This confirms that pregnant women and women who may become pregnant need to be considered during drug development, while being protected as much as possible.

In contrast, the main factors that can lead to proscribing the use of a drug during pregnancy are:

- Clear evidence of risk in humans (data from previous studies and literature)
- Possibility of using therapeutic alternatives, or to avoid or postpone treatment
- Unavailability of safe abortion.

Even in the case of proven harmful effects on fetuses, some drugs may not be fully contraindicated during pregnancy where there is major maternal benefit and availability of safe abortion or where there is potential “loss of chance” for both mother and fetus, meaning that if the maternal disease is not treated effectively during pregnancy it will be detrimental to either or both the mother and child's health.

The drug transfer into human milk can be determined based on concentration of the active substance or metabolites in milk in animal and human studies. This supports recommendations to continue breastfeeding or to continue treatment but discontinue breastfeeding, which is often a very difficult decision in LMIC [34].

In this evolving landscape, the decision to include or not include WoSuPs is not clear-cut. At present, sponsors have to establish inclusion and exclusion criteria without the help of a clear decision tree. The next section proposes a structured and comprehensive framework for the evaluation of the pros and cons of including WoSuPs, based on a combination of data and expert advice.

### DND*i*’s proposal and ethical arguments

To remedy the lack of guidelines for the various situations mentioned above, we present DND*i*'s proposed position on the inclusion of WoSuPs in trials in LMICs and timing during the development plan.

1. First and foremost, DND*i* recommends evaluating the implications of conducting research and assessing benefit and risk of WoSuP inclusion at regular intervals during the drug’s entire life cycle.
2. It will be kept in mind that the exclusion of WoSuP may deprive them of a chance to access new potential treatments or cures of life-threatening diseases for which there is no existing safe and effective treatment.
3. It is proposed that instead of limiting WoSuP inclusion in the trials, a systematic and appropriate benefit and risk assessment is carried out with high scientific and ethical standards.
4. The inclusion of WoSuP will be discussed in light of the severity of the disease in women, the effect of disease on the fetus, the available nonfoetotoxic, nonteratogenic therapeutic alternatives, access to effective contraception, access to safe abortion, the risk of pregnancy, and, should it occur during the trial, the status and progress of that pregnancy.
5. While the benefits of participating in research cannot be underestimated, the use of any investigational drug in this population may pose a risk to the potential fetus, and this risk needs to be mitigated: A negative pregnancy test at inclusion and the provision of effective contraception do not guarantee that a pregnancy will not occur during a trial. It will always be necessary to weigh in the trial benefits, risks, and consequences in a population in which effective contraception is uncertain, even when given for free to WoSuPs during the trial. Points to consider include issues with good understanding of and adherence to contraception, acceptability of possible adverse effects of the contraception, and cultural, personal, and societal issues relating to the absence or postponement of pregnancy during the trial, especially for long duration trials.

#### DNDi’s ethical arguments for including WoSuP

DND*i* studies and develops treatments for life-threatening NTDs that often affect WoSuP. WoSuP are a dynamic subset of the adult and adolescent female population who may need drugs and biologics. DND*i* will always consider whether, when, and how to include WoSuPs in drug development [7].

Before undertaking any and all research, DND*i*, along with investigators and industrial sponsors, will make every effort to ensure that:

- the research corresponds to the health needs and priorities of the population or community in which it is to be carried out
- any intervention or product developed, and knowledge generated, is made available as far as possible for the benefit of that population or community [13]

In those parts of the world with a high prevalence of NTDs, women are particularly vulnerable to harm during research because of their social and cultural conditioning to submit to authority, ask fewer questions, and tolerate pain and suffering. Refusing to include young women in clinical trials further limits their access to treatment and increases their vulnerability to the disease. DND*i* as study sponsor, the IRB and REC, and the investigators analyze carefully such situations and implement strong and traceable follow-up of impact mitigation in this vulnerable population [13].

The assessment algorithm for inclusion of WoSuPs in various phases of drug development is described in Fig 1. To facilitate the analysis, it is recommended that a collaborative “prereview” of the ethical oversight is set-up, conducted by an ad hoc ethics committee including representatives from the target country or countries and from mature countries, in collaboration with internationally recognized scientific experts [41].

Where there is no prior use of the drug in pregnant women, the decision of whether to include WoSuP will be informed by the animal toxicology profile. Where effects have been detected in animals, results must be interpreted carefully because the predictability to humans of positive animal findings is not straightforward. For example, it may be difficult to interpret differences between malformations and simple variations, absence of interspecies reproducibility, or differences in levels of exposures [42].

In the case of long treatment exposure during a clinical trial, repeated evaluation of the benefit and risk of treatment continuation should be made. In the case of unexpected pregnancy, the benefit and risk of treatment continuation versus safe abortion should be evaluated. If the trial was double-blinded, then the blind should be broken to make the exposure assessment.

#### DND*i*’s ethical arguments for retaining WoSuP in the trial if they become pregnant

**Fig 2:**
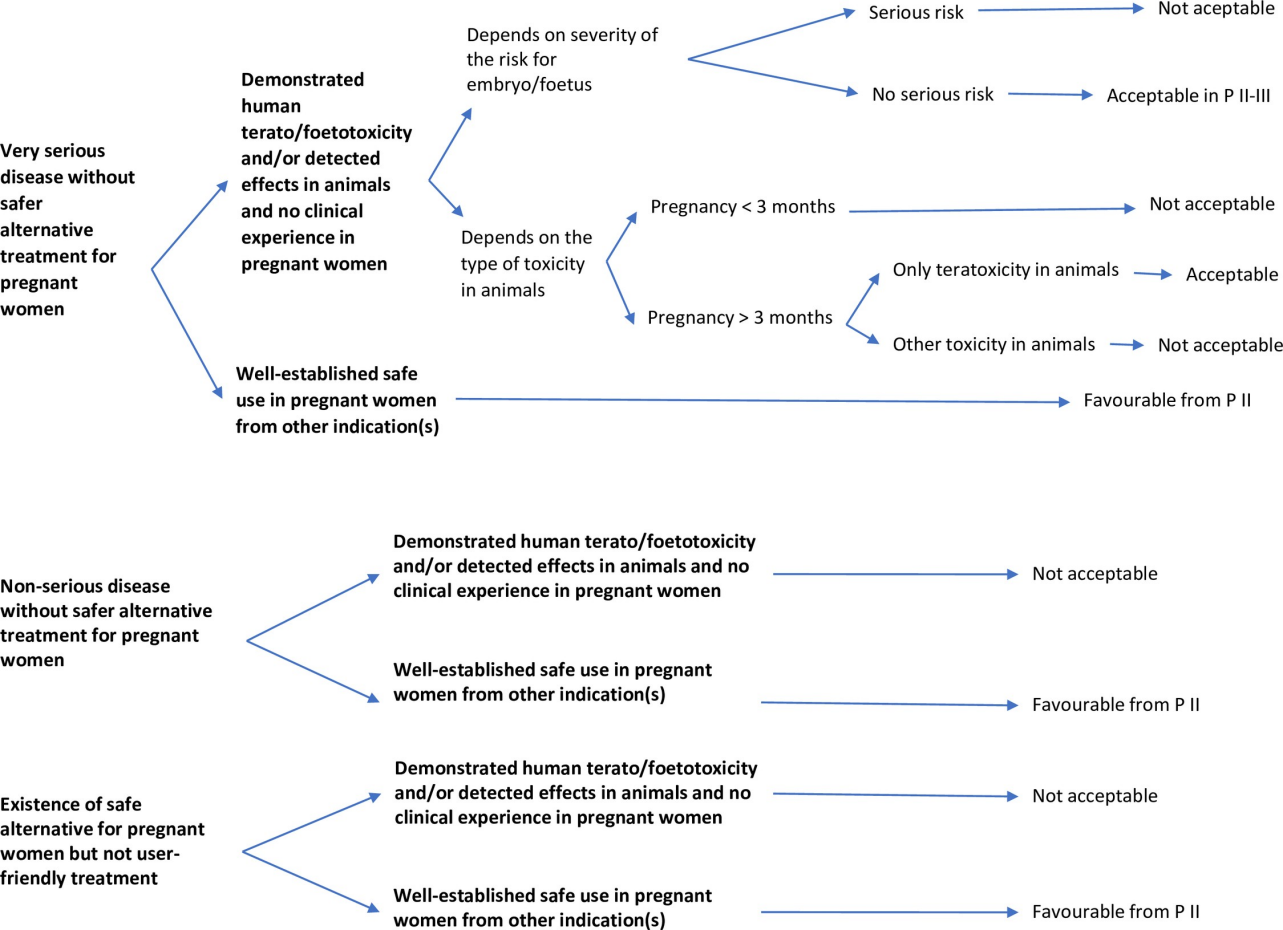
Continuation of clinical trials in women who are unexpectedly detected or become pregnant after inclusion. P, Phase.

The issues for WoSuPs who become pregnant during a trial are summarized in Fig 2. Where the risks of the drug are well-known in animals and humans and/or where there is no clinical experience in pregnant women, it may be acceptable to retain a pregnant woman in a clinical trial, and the decision must be evaluated balancing the risk of death or serious complications from untreated disease. Furthermore, even if the drug is teratogenic but not foetotoxic, pregnant women could be kept in the study after the first trimester. The type of fetal impact must also be considered.

If the use of the drug in pregnant women is well established in other indications, pregnant women could be kept in clinical trials.

In case of exposure in a trial to a new drug with potential teratogenicity or other type of toxicity, safe abortion should be available and offered to WoSuP. Treatment with the test drug can be continued after an abortion.

## Discussion

The above algorithms are proposed to facilitate decision-making about whether to include and retain WoSuPs in clinical trials. The main situations described in these algorithms are based on DND*i* experience of trials.

Inclusion of WoSuP in Phase I and II clinical trials for the treatment of NTDs should be considered carefully, based on evidence available from animal studies, toxicology, and pharmacology studies. The data required before initiation of trials with WoSuPs are defined in the ICH and generally include reproduction toxicity and carcinogenicity data and available human safety data, but the package will depend on what is already known about the product, its type, and indication.

Feasibility studies will increase the chances of completing the trial by informing about recruitment capacity, for example.

Adaptive designs allow the design of randomized trials to be modified while they are ongoing, based on predefined criteria of success or futility [36]. Such designs have been proposed to increase the efficiency of randomized clinical trials while reducing costs and enhancing the likelihood of finding the true benefit, if one exists, of the therapy being studied. Adaptive design refers to making prospectively planned changes to the future course of an ongoing trial on the basis of an analysis of accumulating data from the trial itself. This allows, for example, discontinuation of a dose in a multidose comparative trial or increase the sample size to increase the trial power. Regulators scrutinize such trials and recommend seeking confirmation of the design prior to the trial taking place. Such designs can be used for trials involving WoSuPs for NTDs.

DND*i* intends to implement these algorithms in its development plans, evaluate their applicability, and improve them. Several metrics will be followed up by DND*i*, including the number of WoSuPs included, the number of unexpected pregnancies and their follow-up, and the number of questions received from IRB and RECs. A new version of these algorithms will be updated after the first implementation period.

## Conclusion

The current situation in which WoSuPs are most of the time excluded from clinical trials is unethical because it prevents young women from participating in research and potentially benefitting from investigational new drugs and marketed products. It could also be considered as scientifically unjustifiable to exclude these women, as their metabolism differs from men’s, with different pharmacodynamics and pharmacokinetics, resulting in a dearth of evidence for appropriate dosage for women, even if the dosages of medications are generally based on large clinical trials in which aspects such as weight and age are mainly considered. When a clinical trial represents the only access to an experimental therapy expected to be effective and safe for a life-threatening condition, it is obviously imperative to include young women as well and obtain robust data in this population. This paper is published to encourage other teams developing new drugs in LMIC to test the algorithms and provide feedback.

The landscape and ethical considerations are evolving, and this proposal is our contribution to accelerate change. DND*i* strives to study its drugs against NTDs in WoSuPs in a scientific and ethical manner, in order to provide these women with the same chances of being treated as other populations in LMIC.
